# Hidradenitis suppurativa with systemic autoinflammatory features in patients of Moroccan origin: case report and implications for personalized medicine

**DOI:** 10.3389/fimmu.2026.1775443

**Published:** 2026-03-20

**Authors:** Jérôme Lurel, Fadwa El Aissoug, Fatima-Zahra Hammoud, Dillon Mintoff, Farida Benhadou

**Affiliations:** 1 Department of Dermatology, Hôpitaux Universitaires de Génève (HUG), Geneva, Switzerland; 2 Department of Dermatology, Notre Dame de Grâce, Gosselies, Université Libre de Bruxelles (ULB), Rabat, Belgium; 3 Department of Dermatology, Mohamed V University of Rabat, Ibn Sina University Hospital, Rabat, Morocco; 4 Department of Dermatology, Mater Dei Hospital, Msida, Malta; 5 Department of Dermatology, Hôpital Universitaire de Bruxelles (H.U.B.), Centres Universitaires de Bruxelles (CUB) Hôpital Erasme, Université libre de Bruxelles (ULB), Brussels, Belgium; 6 European Hidradenitis Suppurativa Foundation e.V, Dessau, Germany

**Keywords:** autoinflammation, Behçet’s disease, Hidradenitis suppurativa, HLA typing, MEFV, metainflammation, personalized medicine

## Abstract

Hidradenitis suppurativa (HS) is a chronic inflammatory skin disease primarily affecting intertriginous regions, and emerging evidence suggests that systemic autoinflammation (“metainflammation”) contributes to its clinical heterogeneity. We report two cases of HS in patients of Moroccan origin with overlapping autoinflammatory features. The first case involves a 42-year-old man with HS since 2012, Hurley stage II, treated with adalimumab since 2019 and achieving an IHS4-70, who developed Behçet’s disease-like systemic manifestations in 2025; genetic testing revealed a heterozygous *MEFV* variant, and HLA typing included *A02*, *A68*, *B15*, *B45*, *C02*, and *C06*. The second case involves a 40-year-old man with HS since age 22, Hurley stage II, with persistent oral aphthosis; he achieved IHS4-70 on secukinumab in 2025 and responded to adjunctive colchicine for mucosal lesions, with HLA typing showing *B18*, *B51*, *C07*, and *C15*. These cases illustrate the interplay between HLA subtypes, autoinflammatory gene variants, and systemic inflammation in HS, highlighting a potential autoinflammatory HS subtype. Population-specific genetic factors, particularly in North African populations, may influence disease severity, systemic manifestations, and therapeutic response. Taken together, these observations support the concept that HS, in certain genetically predisposed individuals, may represent a systemic autoinflammatory/metainflammatory disease, underscoring the relevance of personalized therapeutic strategies.

## 1 Introduction

Hidradenitis suppurativa (HS) is a chronic, relapsing inflammatory disorder of the follicular unit, historically considered a localized cutaneous disease but increasingly recognized as a systemic autoinflammatory/metainflammatory disorder. HS predominantly affects intertriginous regions, including axillary, inguinal, perineal, and gluteal areas, manifesting as recurrent painful nodules, abscesses, draining fistulas, and scarring. While its cutaneous manifestations are well-characterized, recent studies highlight the role of systemic inflammation, implicating metabolic dysfunction, adipokine imbalance, dysbiosis, and hyperactivation of innate immune pathways in shaping disease severity and systemic comorbidities (1, 2).

The concept of metainflammation, that is chronic, low-grade inflammation sustained by metabolic and immunologic perturbations provides a unifying framework linking HS with cardiometabolic disease, inflammatory bowel disease, and autoinflammatory syndromes (3). At the cellular level, neutrophil and monocyte hyperactivation, Interleukin (IL)-1β, Tumor necrosis factor (TNF)-α, IL-17 overproduction, and dysregulated inflammasome signaling drive both local and systemic inflammatory processes (3–5).

Behçet’s disease (BD), traditionally classified as a variable-vessel vasculitis, shares overlapping immunopathogenic features, including neutrophil hyperreactivity, excessive IL-1 activity, TNF-α and IL-17 dysregulation, and a strong association with HLA-B51 (6–8). Recent genetic and immunologic studies support its classification as an autoinflammatory disorder, highlighting shared innate immune mechanisms with HS. Although co-occurrence of HS and BD is uncommon, evidence indicates convergence on inflammasome-regulating genes such as *MEFV*, *NLRP3*, *TNFAIP3*, and *PSTPIP1*, suggesting a continuum rather than discrete entities (9–11).

This report is novel in highlighting this intersection in patients of Moroccan origin, a population with high prevalence of HLA-B51 and potential enrichment of autoinflammatory gene variants. The cases are unique because data on Moroccan-origin patients are very limited, and the genetic analysis of *MEFV* variants provides novel insight into autoinflammatory predisposition. By describing HS with concurrent BD-like systemic manifestations, we provide insight into genetic, immunologic, and metainflammatory factors that may define a distinct autoinflammatory HS subtype, emphasizing the relevance of precision medicine approaches targeting both skin and systemic inflammation.

## 2 Cases description

### 2.1 Case 1

**Figure 1:**
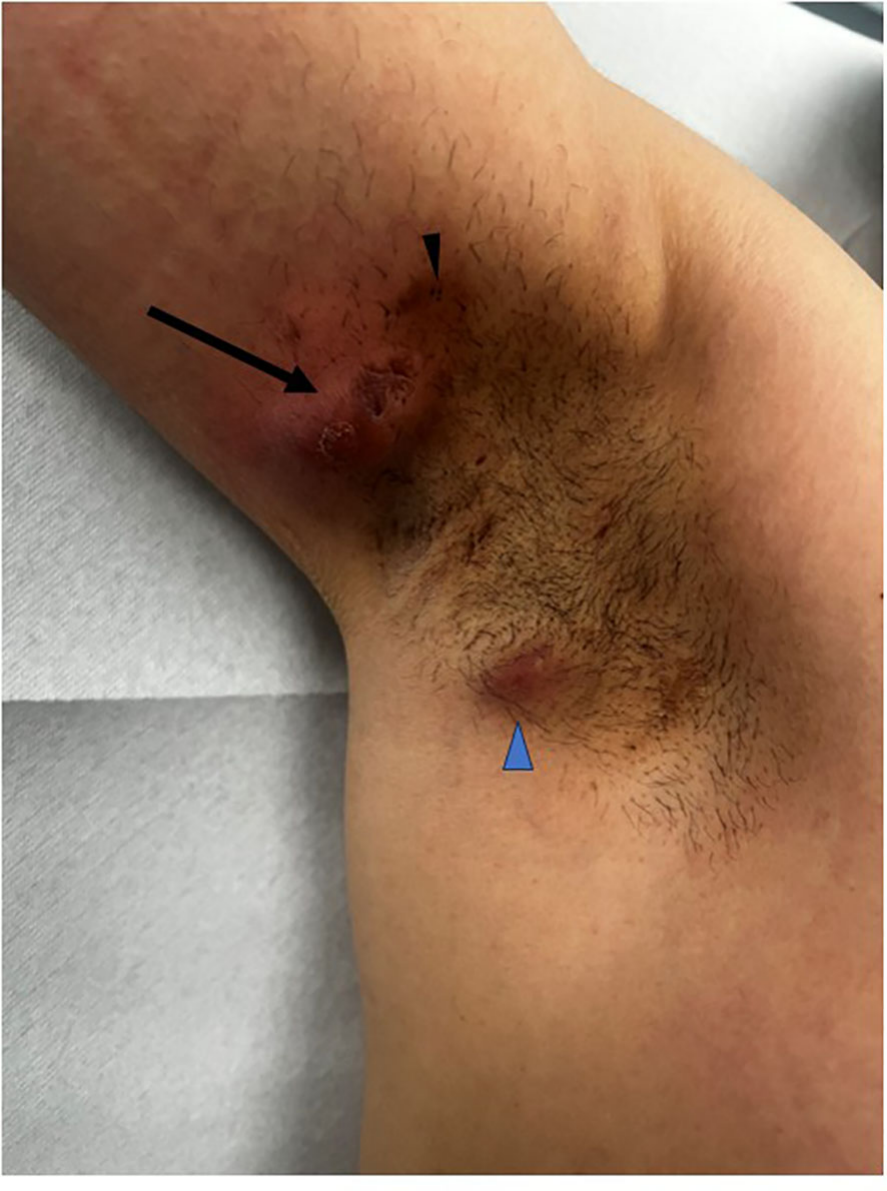
Hidradenitis suppurativa – clinical manifestations. Right axilla: Draining fistula and abscess (black arrow), acute inflammatory abscess (blue arrowhead), and comedones (black arrowhead), characteristic features of HS.

A 42-year-old non-smoking man of Moroccan origin presented to our department in 2016 with recurrent abscesses and draining lesions involving the axillary, inguinal, and perineal regions. The first symptoms had appeared at the age of 18. The patient had no relevant personal or family medical history and no other chronic inflammatory disorders. He worked as a taxi driver. He was born in Morocco and moved to Belgium at the age of 6. The patient had received multiple courses of antibiotics, including tetracyclines and a combination of rifampicin and clindamycin, with only partial disease control. At presentation, lesions consisted predominantly of abscesses and draining fistulas, mainly affecting the axillary regions (**Figure 1**). Inflammatory nodules and residual scarring were observed in the inguinal and perineal areas. The clinical history, chronic course, and the presence of abscesses, inflammatory nodules, comedones, and draining fistulae in typical skin locations supported the diagnosis of HS classified as Hurley stage II with an IHS4 score of 37. The disease had a substantial impact on the patient’s quality of life, as reflected by a HiSQOL score of 45.The patient’s body mass index (BMI) was 25.9 kg/m².

**Figure 2:**
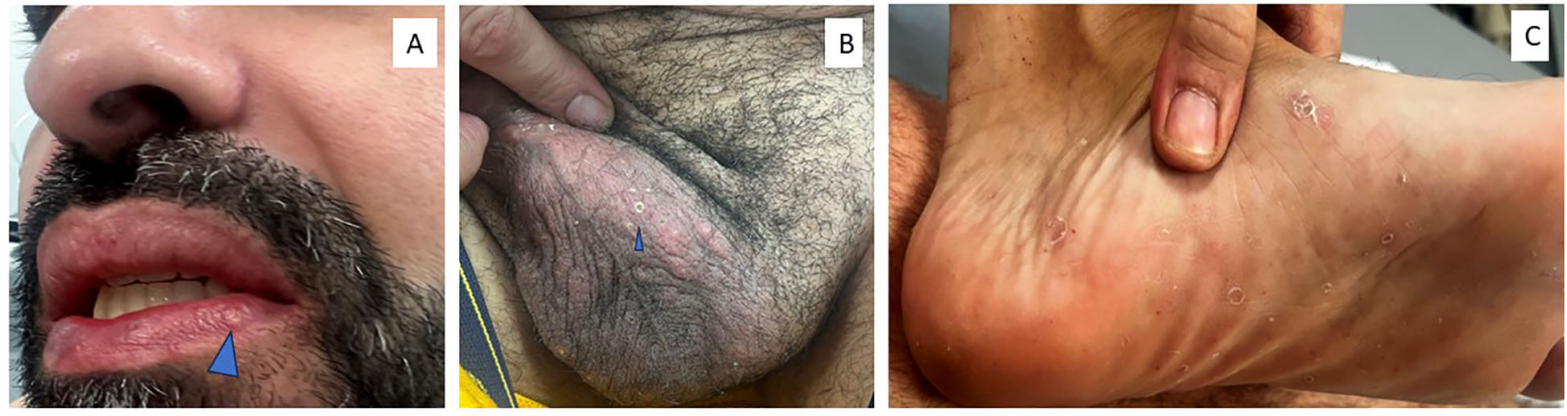
Behçet disease manifestations. **(A)** Oral aphthosis on the lower lip. **(B)** Genital aphthosis: Multiple painful, well-demarcated ulcerations with a necrotic base on the genital mucosa. **(C)** Pustular lesions on left plantar surface.

Due to an insufficient response to antibiotic therapy, adalimumab was initiated in 2019 according to the standard HS induction and maintenance regimen: a loading dose of 160 mg at week 0, followed by 80 mg at week 2, and then 40 mg weekly starting at week 4. Long-term disease control was achieved, with a marked improvement in disease activity, reaching an IHS4 score of 10 after six months and a HiSQOL score of 0. In 2023, adalimumab was discontinued because of perceived disease stability and inconsistent treatment adherence. Approximately one year later, the patient developed systemic inflammatory manifestations, including recurrent oral and genital aphthous ulcers, erythema nodosum of the lower limbs, pseudofolliculitis of the trunk, and painful pustular lesions on the palms and soles, while HS remained clinically quiescent (**Figure 2**). Laboratory investigations revealed marked neutrophilia and elevated C-reactive protein (CRP) levels, with preserved hepatic and renal function. Histopathological examination of a biopsied palmoplantar pustule demonstrated dense neutrophilic folliculitis without evidence of infection. Based on the International Criteria for BD (ICBD) (12) which require recurrent oral ulceration plus at least two of the following features, genital ulceration, skin lesions (erythema nodosum or pustules), ocular involvement, vascular manifestations, or a positive pathergy test, the diagnosis of BD associated to HS was established.

Sequencing of an autoinflammatory disease gene panel identified a heterozygous missense variant in the *MEFV* gene (rs3743930), as detailed in **Table 1** (13, 14). This variant results in a p.Glu148Gln (E148Q) substitution in the pyrin protein, a key regulator of the inflammasome. Although classified as benign/likely benign according to ACMG criteria due to its prevalence in certain populations, its SIFT score (0.02) and CADD score (21) suggest a potential functional impact on protein stability (ΔΔG 0.33 kcal/mol), possibly acting as a genetic modifier of autoinflammatory susceptibility. HLA typing revealed the following alleles: A*02, A*68, B*15, B*45, C*02, and C*06.

**Table 1:** Genetic characteristics of the p.Glu148Gln (E148Q) missense variant in the *MEFV* gene identified in a patient with Hidradenitis Suppurativa.

rsID	rs3743930
Gene	*MEFV*
Transcript	ENST00000570511.5
Chromosome	16
Position	16:3254626-3254626
cDNA position	442
Allele	G
Alternate	C
Zygosity	Heterozygous
Protein Position	148
Amino Acid / Change	E / Q
Protein Effect	Missense
ACMG Classification	Benign
GnomAD AF	0.04
SIFT	0.02 (deleterious)
CADD Score	21
Alpha Missesense Classification	Likely Benign
Metdome Mutation Tolerance	Neutral to variation
Predicted Stability Change (**ΔΔG^Stability)^**	0.33 kcal/mol (stabilising)

The table summarizes the genomic coordinates and in silico pathogenicity predictions for the rs3743930 variant. The *MEFV* gene encodes pyrin, a key protein in the regulation of the inflammasome. While the E148Q substitution is classified as "Benign" or "Likely Benign" by ACMG and AlphaMissense criteria, its relatively high CADD score (21) and SIFT prediction (deleterious) suggest a functional impact. In the context of Hidradenitis Suppurativa), mutations or polymorphisms in the *MEFV* gene are increasingly studied as potential genetic modifiers that may exacerbate autoinflammation and contribute to the follicular occlusion and chronic abscess formation characteristic of the disease.

**Figure 3:**
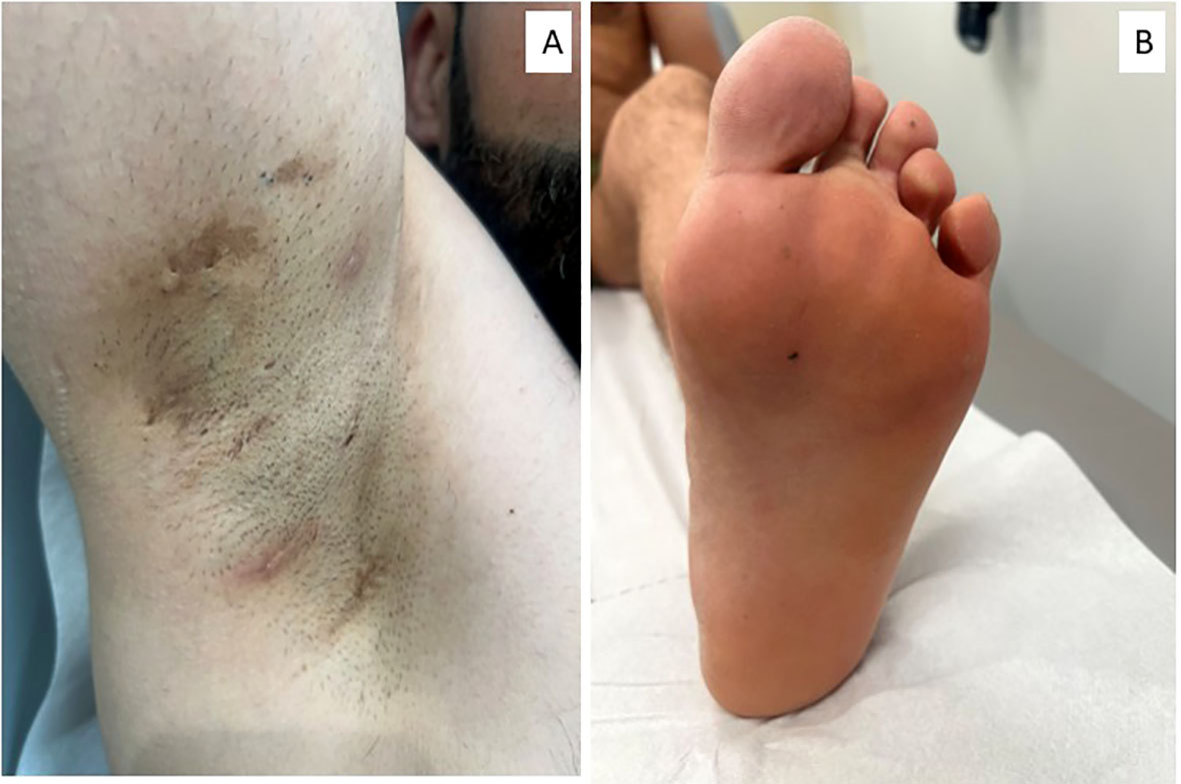
Improvement of HS lesions three months after reintroduction of adalimumab. **(A)** Right axilla, showing persistent scarring but no active inflammatory lesions. **(B)** Left plantar surface with resolution of pustular lesions.

Reintroduction of adalimumab at a dose of 40 mg weekly led to rapid clinical and biochemical remission, with complete resolution of mucocutaneous lesions and normalization of systemic inflammatory markers within a few weeks (IHS4 = 0; HiSQOL = 0) (**Figure 3**). This response underscores the central role of TNF-mediated pathways in this overlapping autoinflammatory phenotype. The patient has since remained in sustained remission, with no recurrence of HS lesions or BD-related mucocutaneous flares under continued weekly adalimumab therapy.

### 2.2 Case 2

A 40-year-old man of Moroccan origin, born in Belgium, presented with recurrent abscesses and nodules affecting the gluteal, axillary, and pubic regions. The first cutaneous symptoms occurred at the age of 22, and he had a history of recurrent oral aphthosis since the age of 18. He had no relevant personal or family medical history, was a non-smoker, and had no history of acne. Initial treatment with standard HS antibiotic therapies, including oral tetracyclines and a combination of rifampicin and clindamycin, failed to provide significant clinical improvement. Moreover, oral aphthous lesions worsened during this period. At the time of consultation, the patient presented with multiple abscesses involving the axillary and gluteal regions and painful oral aphthosis. Based on the chronic disease course and the presence of typical HS lesions, a diagnosis of HS was established, classified as Hurley stage II with an IHS4 score of 50. The patient’s BMI was 26.8 kg/m². Laboratory investigations were unremarkable. The disease had a substantial impact on the patient’s quality of life, as reflected by a HiSQOL score of 33. Given the highly inflammatory phenotype and the patient’s unwillingness to receive TNF inhibitor therapy, secukinumab was initiated according to the standard HS dosing regimen: 300 mg subcutaneously at weeks 0, 1, 2, 3, and 4, followed by 300 mg every 4 weeks.

HS lesions responded favorably, with a reduction of the IHS4 score to 9 after three months of treatment, and a complete normalization of quality of life, with a HiSQOL score of 0. However, recurrent oral aphthosis persisted, indicating that these BD-like manifestations were not adequately controlled by IL-17 inhibition. Oral aphthosis was subsequently treated with colchicine at a dose of 1 mg daily, resulting in marked clinical improvement.

Since the initiation of colchicine, the patient has remained in sustained remission of BD-like oral lesions, while HS disease control has been maintained under continued secukinumab therapy. HLA typing revealed the presence of *B18*, *B51*, *C07, and C*15 alleles; genetic testing was declined by the patient. Given this incomplete clinical presentation, a diagnosis of HS associated with BD-like lesions was ultimately retained.

## 3 Discussion

### 3.1 HLA subtypes and genetic background

Both patients carried HLA class I alleles linked to autoinflammatory and immune-mediated disorders, consistent with immunogenetic data. HLA-B51 is a well-established risk factor for BD, present in 55–63% of patients from Mediterranean and North African populations versus 17–22% of controls, corresponding to an odds ratio of ~5.8. This allele’s prevalence along the historical “Silk Road” indicates population-specific contributions to autoinflammatory phenotypes (7).

Beyond HLA, variants in autoinflammatory genes such as *MEFV* which encodes the inflammasome regulator pyrin have been identified in both HS and BD cohorts and may contribute to disease severity and systemic manifestations (9). The observed clinical overlap between HS and systemic inflammatory conditions reflects a growing recognition that HS can manifest as part of a broader autoinflammatory spectrum. A key element of this spectrum is the association with Familial Mediterranean Fever (FMF), the most prevalent monogenic autoinflammatory disease (9).

In our case 1, the identification of the heterozygous *MEFV* missense variant rs3743930 (p.Glu148Gln) is highly significant. Historically, *MEFV* mutations were strictly linked to FMF. However, recent evidence suggests that variants like E148Q act as potent disease modifiers in HS, even when classified as ‘benign’ by traditional ACMG criteria. Despite its relatively high frequency in Mediterranean populations, the CADD score of 21 and SIFT prediction of 0.02 (deleterious) for this specific variant support its role in altering pyrin function. Studies have shown that HS patients carry *MEFV* mutations at a significantly higher frequency than the general population, and these carriers often exhibit more severe HS phenotypes, higher systemic inflammatory markers (CRP), and a greater burden of neutrophilic dermatoses (9–11).

This association suggests that *MEFV*-associated HS represents a specific autoinflammatory endotype. In these patients, the “primed” pyrin inflammasome lowers the threshold for IL-1β release in response to follicular occlusion or metabolic DAMPs. This explains the “FMF-like” systemic flares observed in Case 1, such as erythema nodosum and aphthosis, which were triggered during a period of HS quiescence. Furthermore, this genetic link provides a strong pharmacological rationale for the use of colchicine (Case 2) which specifically targets pyrin-mediated inflammation, providing a bridge between the management of FMF and severe, syndromic HS.

### 3.2 Metainflammation and the systemic dimension of HS

Both cases demonstrated systemic inflammatory features, consistent with the emerging concept of “metainflammation”, a state of chronic, low-grade systemic inflammation driven by metabolic and immunologic perturbations. Unlike acute inflammation, metainflammation in HS extends beyond the follicular unit, involving a complex interplay between adipose tissue activity, the gut-skin axis, and innate immune dysregulation (15).

Even in patients with a moderate BMI, such as those described here (25.9 and 26.8 kg/m²), the role of metabolic “second signals” should not be overlooked. Adipose tissue in HS acts as an active endocrine organ, secreting pro-inflammatory adipokines (e.g., leptin, resistin) while downregulating anti-inflammatory factors like adiponectin (14). These metabolic triggers can act as endogenous Damage-Associated Molecular Patterns (DAMPs), lowering the activation threshold of the NLRP3 and pyrin inflammasomes. This synergizes with the patients’ underlying genetic susceptibility (*MEFV* variants or HLA-B51) to fuel a “vicious cycle” of IL-1β and TNF-α production, explaining the systemic spread of inflammation (15).

In Case 1, the development of systemic manifestations during a period of HS remission is particularly revealing. This “clinical discordance” suggests that while adalimumab successfully suppressed the local follicular TNF-α cascade, the underlying metainflammatory drive remained active, manifesting through different target organs (mucosa and distal skin). This supports the hypothesis that systemic autoinflammatory pathways can be activated independently of active cutaneous suppuration. Case 2 further illustrates this complexity through the persistence of mucosal aphthosis despite excellent control of skin lesions under secukinumab. This suggests a divergent cytokine requirement for different tissue compartments: while IL-17A is a primary driver of neutrophilic abscesses in HS, the BD-like mucosal manifestations may be more dependent on the IL-1β axis or neutrophil chemotaxis, which responded only after the addition of colchicine (15, 16).

These observations highlight the necessity of addressing HS not merely as a localized skin disease, but as a multi-organ inflammatory syndrome. The therapeutic success observed, either through TNF α blockade or the stabilization of the inflammasome with colchicine, underscores the value of targeting the broader metainflammatory milieu rather than purely lesion-directed approaches.

### 3.3 Syndromic and monogenic associations

The observed clinical overlap between HS and systemic inflammatory conditions in our cases reflects a growing recognition in the literature that HS can present not only as an isolated cutaneous disorder but also as part of a broader syndromic autoinflammatory spectrum. Classical examples of syndromic HS include combinations of pyoderma gangrenosum, acne and suppurative hidradenitis (PASH), pyogenic arthritis, pyoderma gangrenosum, acne and HS (PAPASH), as well as associations with synovitis, acne, pustulosis, hyperostosis, and osteitis (SAPHO) and related constellations, all of which share underlying innate immune dysregulation and neutrophil-driven inflammation (17–20).

Beyond these dermatologic syndromes, HS has been reported in association with monogenic autoinflammatory diseases, most notably familial FMF, which shares pyrin inflammasome dysregulation and IL-1β–mediated pathways with HS. Population-based analyses demonstrate an increased prevalence of FMF among HS patients compared with controls, and complex HS phenotypes are more frequently observed in carriers of pathogenic *MEFV* variants, suggesting a genetic predisposition to systemic inflammation that modulates HS severity (21). Related case reports have also described HS in the context of other monogenic autoinflammatory syndromes such as hyper-IgD syndrome (HIDS), implicating metabolic inflammasome pathways in both cutaneous and systemic manifestations (22). These associations support the notion proposed by several reviews that syndromic HS represents different faces of a shared autoinflammatory continuum, wherein genetic variants (e.g., *MEFV*, *PSTPIP1*, *NOD2*, *NCSTN*) and dysregulated cytokine networks (IL-1β, TNF-α, IL-17) contribute to phenotypic diversity and systemic involvement beyond the skin (19).

### 3.4 Toward a novel autoinflammatory HS subtype

We propose a subclassification of Sporadic HS: largely influenced by environmental and metabolic factors, Familial HS: associated with gamma-secretase mutations and Syndromic autoinflammatory HS: co-occurrence with systemic immune dysregulation and overlapping autoinflammatory phenotypes, potentially including BD-like features (23). The distinction between inherited and familial HS is thus subtle, but significant. The term “inherited” implies the direct transmission of specific deleterious genetic variants from parent to offspring, as seen in monogenic forms of HS. In contrast, “familial” HS denotes cases with a familial aggregation of disease without a singular genetic determinant, often reflecting polygenic inheritance or shared environmental exposures. Syndromic HS warrants its own classification, given the distinct systemic implications and management challenges associated with these cases (2’,^24^).

This classification is pathogenetically grounded, informs genotype-phenotype correlation, and guides precision therapeutic strategies targeting both skin and systemic inflammation.

### 3.5 Precision medicine and the metainflammatory paradigm: a clinical shift

The integration of HLA typing and autoinflammatory genetic profiling into clinical practice represents a fundamental shift from a “one-size-fits-all” approach toward precision medicine in HS management. This strategy is particularly crucial for North African populations, where the convergence of specific HLA alleles (e.g., B51) and *MEFV* variants creates a unique “immunogenetic signature” that may predispose patients to more aggressive, syndromic, or “Behçetoid” phenotypes.

**Figure 4:**
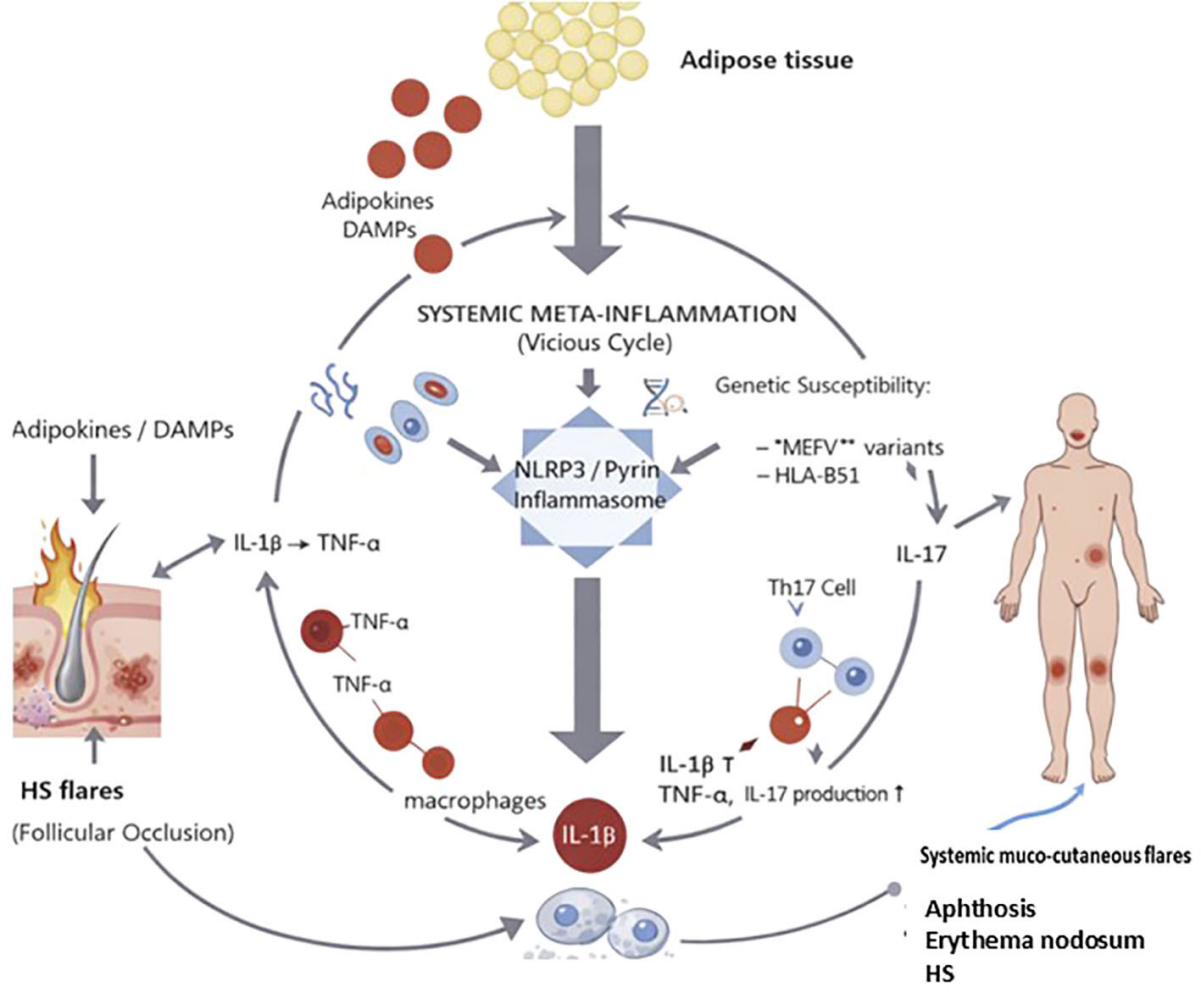
The vicious cycle of metainflammation in Hidradenitis suppurativa. Adipose tissue acts as an endocrine organ, releasing pro-inflammatory adipokines (Damage-Associated Molecular Patterns (DAMPs) that lower the activation threshold of the NLRP3 and pyrin inflammasomes. This metabolic drive, combined with genetic susceptibility (Mediterranean Fever gene (*MEFV*) variants, HLA-B51), fuels a systemic “vicious cycle” of IL-1β, TNF-α, and IL-17 production. This mechanism explains the systemic spread of inflammation and the occurrence of mucocutaneous flares (e.g., aphthosis, erythema nodosum) even when localized cutaneous HS lesions are quiescent.

Our cases illustrate that HS is not merely a localized follicular disorder but a systemic metainflammatory condition. Even in patients with a moderate BMI, adipose tissue acts as an active endocrine organ, secreting pro-inflammatory adipokines that function as endogenous DAMPs. These metabolic signals lower the activation threshold of the NLRP3 and pyrin inflammasomes, exacerbating the underlying genetic susceptibility. This “vicious cycle” of IL-1β, TNF-α, and IL-17 production explains the systemic spread of inflammation, where mucocutaneous flares can occur even when cutaneous lesions are quiescent (**Figure 4**).

### 3.6 Early stratification and targeted therapy

Genetic screening facilitates early risk stratification, allowing clinicians to identify patients at risk for extra-cutaneous complications such as uveitis, arthritis, or amyloidosis before they become debilitating. Furthermore, this approach guides tailored cytokine targeting: TNF-α blockade remains the cornerstone for “overlapping” phenotypes (HS/BD), addressing both vascular and follicular inflammation, IL-17A inhibition offers superior cutaneous control but may require adjunctive therapy, such as colchicine, to stabilize the pyrin inflammasome and manage mucosal or neutrophilic manifestations that escape IL-17 blockade. The persistent mucosal involvement in Case 2 despite IL-17A inhibition aligns with recent clinical data suggesting that while the IL-17 pathway is involved in BD pathogenesis, TNF-α blockade remains superior for controlling mucocutaneous and vascular manifestations (24–26).

In this precision medicine model, the goal of therapy evolves from simple “lesional clearance” to complete biochemical and systemic remission. By monitoring systemic inflammatory markers alongside genetic profiles, clinicians can optimize long-term outcomes and mitigate the cardiovascular and metabolic risks inherent to the HS-autoinflammatory continuum. This holistic, genotype-phenotype approach moves HS management beyond purely symptomatic care toward a proactive, patient-specific therapeutic model.

## 4 Conclusion

These cases highlight that HS can extend beyond a localized cutaneous disorder, presenting as part of a broader systemic autoinflammatory/metainflammatory spectrum in genetically predisposed individuals. The coexistence of HLA risk alleles, autoinflammatory gene variants such as *MEFV*, and systemic inflammatory features may define a distinct HS subtype with overlapping BD-like manifestations. The potential role of *MEFV* variants as an immunogenetic signature deserves particular attention, as these variants may not only confer susceptibility but also influence disease severity and systemic expression. This hypothesis supports the concept that *MEFV*-associated inflammasome dysregulation could contribute to defining a genetically and clinically distinct HS phenotype within the autoinflammatory spectrum.

Importantly, distinguishing between “classic” HS and HS-like lesions associated with systemic autoinflammation is often challenging. Features such as abscesses, fistulas, may overlap with cutaneous manifestations secondary to autoinflammatory dysregulation, and the presence of mucosal or systemic flares (e.g., aphthosis, erythema nodosum) may help identify HS associated with a BD-like phenotype. In some patients, as illustrated by Case 2, it is more accurate to describe the systemic component as BD-like, reflecting an incomplete or atypical presentation rather than classical BD disease. This underscores that the combination of HS and systemic autoinflammatory features can exist along a continuum, rather than as two strictly separate entities.

These findings have important implications for clinical practice. Recognition of overlapping autoinflammatory HS phenotypes informs early risk stratification, therapeutic selection, and monitoring, emphasizing that treatment should target both cutaneous lesions and systemic inflammatory pathways. However, several limitations should be acknowledged: these are case reports from a limited number of patients, which restricts generalizability; genetic analyses were incomplete in Case 2, limiting conclusions about underlying predisposition; and long-term outcomes beyond the observed follow-up remain unknown. Despite these limitations, these cases reinforce the concept that HS is not solely a skin disease but can manifest as a multifaceted autoinflammatory syndrome, in which systemic inflammation may occur independently of active cutaneous lesions. Future studies are needed to clarify diagnostic criteria for HS with BD-like or autoinflammatory features, explore the prevalence of associated genetic variants, and optimize targeted therapeutic strategies for this emerging HS subtype.

## Data Availability

The original contributions presented in the study are included in the article/supplementary material. Further inquiries can be directed to the corresponding author.

## References

[1] Vossen ARJV van der Zee HH Prens EP . Hidradenitis suppurativa: a systematic review integrating inflammatory pathways into a cohesive pathogenic model. Front Immunol. (2018) 9:2965. doi: 10.3389/fimmu.2018.02965. PMID: 30619323 PMC6302105

[2] D’Onghia M Malvaso D Galluccio G Antonelli F Coscarella G Rubegni P . Evidence on hidradenitis suppurativa as an autoinflammatory skin disease. J Clin Med. (2024) 13:5211. doi: 10.3390/jcm13175211. PMID: 39274425 PMC11396593

[3] Mintoff D Agius R Benhadou F Das A Frew JW Pace NP . Obesity and hidradenitis suppurativa: targeting meta-inflammation for therapeutic gain. Clin Exp Dermatol. (2023) 48:984–90. doi: 10.1093/ced/llad182. PMID: 37171791

[4] Garcovich S Genovese G Moltrasio C Malvaso D Marzano AV . PASH, PAPASH, PsAPASH, and PASS: the autoinflammatory syndromes of hidradenitis suppurativa. Clin Dermatol. (2021) 39:240–65. doi: 10.1016/j.clindermatol.2020.10.016. PMID: 34272017

[5] Kuiper JJ Prinz JC Stratikos E Kuśnierczyk P Arakawa A Springer S . EULAR study group on ‘MHC-I-opathy’: identifying disease-overarching mechanisms across disciplines and borders. Ann Rheum Dis. (2023) 82:887–96. doi: 10.1136/ard-2022-222852. PMID: 36987655 PMC10313995

[6] Takeuchi M Kastner DL Remmers EF . The immunogenetics of Behçet’s disease: a comprehensive review. J Autoimmun. (2015) 64:137–48. doi: 10.1016/j.jaut.2015.08.013. PMID: 26347074 PMC4628864

[7] Burillo-Sanz S Montes-Cano MA García-Lozano JR Olivas-Martínez I Ortego-Centeno N García-Hernández FJ . Behçet’s disease and genetic interactions between HLA-B*51 and variants in genes of autoinflammatory syndromes. Sci Rep. (2019) 9:2831. doi: 10.1038/s41598-019-39113-5. PMID: 30808881 PMC6391494

[8] Hughes T . HLA-B51 and Behçet’s disease: an updated review. Front Immunol. (2020) 11:1234. doi: 10.3389/fimmu.2020.01234. PMID: 32625213 PMC7311670

[9] Vural S Hatemi G Alpdoğan T . Higher frequency of MEFV gene variants in patients with hidradenitis suppurativa compared with healthy controls. JAMA Dermatol. (2019) 155:1193–5 41574757

[10] Marzano AV . Autoinflammatory genes and HS: MEFV mutations in severe disease. Front Immunol. (2019) 10:2156. doi: 10.3389/fimmu.2019.02156. PMID: 31572374 PMC6753178

[11] Livneh A . Familial Mediterranean fever: genetics and autoinflammatory implications. Front Immunol. (2017) 8:1404. doi: 10.3389/fimmu.2017.01404. PMID: 29123528 PMC5662558

[12] International Team for the Revision of the International Criteria for Behçet’s Disease . The International Criteria for Behçet’s Disease (ICBD): a collaborative study of 27 countries on the sensitivity and specificity of the new criteria. J Eur Acad Dermatol Venereol. (2014) 28:338–47. doi: 10.1111/jdv.12107. PMID: 23441863

[13] Wiel L Baakman C Gilissen D Veltman JA Vriend G Gilissen C . MetaDome: Pathogenicity analysis of genetic variants through aggregation of homologous human protein domains. Hum Mutat. (2019) 40:1030–8. doi: 10.1002/humu.23798. PMID: 31116477 PMC6772141

[14] Rodrigues CHM Pires DEV Ascher DB . DynaMut2: Assessing changes in stability and flexibility upon single and multiple point missense mutations. Protein Sci. (2021) 30:60–9. doi: 10.1002/pro.3942. PMID: 32881105 PMC7737773

[15] Mintoff D Benhadou F Pace NP Frew JW . Metabolic syndrome and hidradenitis suppurativa: epidemiological, molecular, and therapeutic aspects. Int J Dermatol. (2022) 61:1175–86. doi: 10.1111/ijd.15910. PMID: 34530487

[16] Moran B Sweeney CM Hughes R Malara A Kanni T van der Zee HH . Hidradenitis suppurativa is characterized by dysregulation of the Th17:Treg cell axis, which is corrected by anti-TNF therapy. J Invest Dermatol. (2017) 137:2389–95. doi: 10.1016/j.jid.2017.05.033. PMID: 28652108

[17] Cugno M Borghi A Marzano AV . PAPA, PASH and PAPASH syndromes: pathophysiology, presentation and treatment. Am J Clin Dermatol. (2017) 18:555–62. doi: 10.1007/s40257-017-0265-1. PMID: 28236224

[18] Maronese CA Moltrasio C Marzano AV . Hidradenitis suppurativa-related autoinflammatory syndromes: an updated review on the clinics, genetics, and treatment of Pyoderma gangrenosum, Acne and Suppurative Hidradenitis (PASH), Pyogenic Arthritis, Pyoderma gangrenosum, Acne and Suppurative Hidradenitis (PAPASH), Synovitis, Acne, Pustulosis, Hyperostosis and Osteitis (SAPHO), and rarer forms. J Clin Med. (2023) 12:207. doi: 10.3390/jcm12010207. PMID: 38423685

[19] Marzano AV Trevisan V Gattorno M Ceccherini I De Simone C Crosti C . Pyogenic arthritis, pyoderma gangrenosum, acne, and hidradenitis suppurativa (PAPASH): a new autoinflammatory syndrome associated with a novel mutation of the PSTPIP1 gene. JAMA Dermatol. (2013) 149:762–4. doi: 10.1001/jamadermatol.2013.2907. PMID: 23571383

[20] Pace N Mintoff D Borg I . The genomic architecture of hidradenitis suppurativa—a systematic review. Front Genet. (2022) 13:861241. doi: 10.3389/fgene.2022.861241. PMID: 35401657 PMC8986338

[21] Guillem P Mintoff D Kabbani M Cogan E Vlaeminck-Guillem V Duquesne A . Case report: comorbid hyper-IgD syndrome and hidradenitis suppurativa – a new syndromic form of HS? A report of two cases. Front Immunol. (2022) 13:883811. doi: 10.3389/fimmu.2022.883811. PMID: 35720358 PMC9204359

[22] Mintoff D Pace NP . Precision in terminology: classifying genetic influence in hidradenitis suppurativa. Arch Dermatol Res. (2025) 317:780. doi: 10.1007/s00403-025-04284-x. PMID: 40411630

[23] Mintoff D Pace NP Borg I . Management of patients with hidradenitis suppurativa having underlying genetic variation: a systematic review and a call for precision medicine. Clin Exp Dermatol. (2023) 48:67–72. doi: 10.1093/ced/llac045. PMID: 36630659

[24] Sadeghi A Rostamzad P Najari S Mahmoudi M Rezaei N . Immunopathogenesis of Behçet’s disease with special focus on Th17 cells and Th17-related cytokines. Front Immunol. (2021) 12:645251

[25] Tong B Liu X Xiao J Liao H . The role of pyrin inflammasome in autoimmune and autoinflammatory diseases. Front Immunol. (2021) 12:664402 41810299

[26] Booty MG Chae JJ Masters SL . The MEFV E148Q variant is not a disease-causing mutation for familial Mediterranean fever. Annals of the Rheumatic Diseases. (2009) 68:995–8.

